# Can diffusion tensor imaging (DTI) outperform standard magnetic resonance imaging (MRI) investigations in post-COVID-19 autoimmune encephalitis?

**DOI:** 10.48101/ujms.v127.8562

**Published:** 2022-05-10

**Authors:** Francesco Latini, Markus Fahlström, David Fällmar, Niklas Marklund, Janet L. Cunningham, Amalia Feresiadou

**Affiliations:** a Neurosurgery, Department of Medical Sciences, Uppsala University, Uppsala, Sweden; b Radiology, Department of Surgical Sciences, Uppsala University, Uppsala, Sweden; c Neurosurgery, Department of Clinical Sciences Lund, Lund University, Skåne University Hospital, Lund, Sweden; d Psychiatry, Department of Medical Sciences, Uppsala University, Uppsala, Sweden; e Neurology, Department of Medical Sciences, Uppsala University, Uppsala, Sweden

**Keywords:** COVID-19, white matter, encephalitis, DTI, MRI

## Abstract

**Background:**

Neurological and psychiatric manifestations related to severe acute respiratory syndrome coronavirus-2 (SARS-CoV-2) infection are widely recognised. Standard magnetic resonance imaging (MRI) investigations are normal in 40–80% of symptomatic patients, eventually delaying appropriate treatment when MRI is unrevealing any structural changes. The aim of this study is to investigate white matter abnormalities during an early stage of post-COVID-19 (coronavirus disease 2019) encephalitis while conventional MRI was normal.

**Methods:**

A patient with post-COVID-19 autoimmune encephalitis was investigated by serial MRIs and diffusion tensor imaging (DTI). Ten healthy control individuals (HC) were utilised as a control group for the DTI analysis. Major projection, commissural and association white matter pathways were reconstructed, and multiple diffusion parameters were analysed and then compared to the HC average using a z-test for serial examinations.

**Results:**

Eleven days after the onset of neurological symptoms, DTI revealed early white matter changes, compared with HC, when standard MRI was normal. On day 68, DTI showed multiple white matter lesions compared with HC, visible at this time also by the MRI images, indicating inflammatory changes in different association and projection white matter pathways.

**Conclusion:**

We confirm a limitation in the sensitivity of conventional MRI at the acute setting of post-COVID-19 autoimmune encephalitis. A complementary DTI investigation could be a valuable diagnostic tool in early therapeutic decisions concerning COVID-19-related neurological symptoms.

## Introduction

Coronavirus disease 2019 (COVID-19) is an on-going viral pandemic caused by the severe acute respiratory syndrome coronavirus-2 (SARS-CoV-2). Neurological and psychiatric manifestations related to SARS-CoV-2 infection are widely reported (1–3). The neuro-invading potential of SARS-CoV-2 is still under debate (4). Standard magnetic resonance imaging (MRI) is able to detect COVID-19-related lesions such as ischemic/haemorrhagic lesions (5–7). In 40–80% of patients with suspected acute stage COVID-19-related neurological symptoms, conventional MRI does not detect structural lesions in the central nervous system (CNS) (3, 7, 8) even when clinically serious neurological focal findings are present (4, 5, 8, 9). Diagnostic difficulties may delay appropriate treatment when MRI is unrevealing (7, 8). Diffusion tensor imaging (DTI) is an advanced neuroimaging technique able to detect microstructural changes in white matter diffusivity (10, 11). DTI is a more sensitive marker of neuropathology than other MRI sequences in a broad spectrum of diseases/conditions, such as concussions (12), tumours (13) and even in early phase of other viral infections such as those caused by Herpes Simplex Virus (HSV) and Human Immunodeficiency Virus (HIV) (14, 15). The only previous study using DTI imaging on COVID-19 patients was performed 3–4 months after recovery (16). To date, no previous study has demonstrated white matter abnormalities during an early stage of post-COVID-19 encephalitis while conventional MRI was normal.

## Materials and methods

### Case: Clinical course

**Figure 1:**
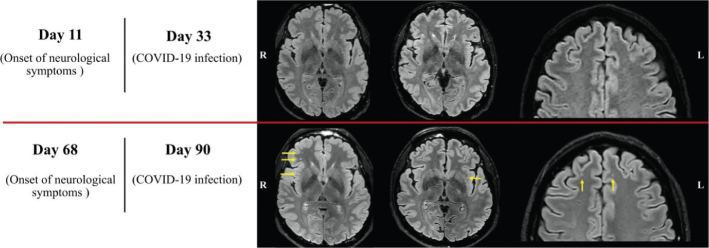
The trend in neuroradiological detection of white matter changes in our patient is summarised. FLAIR images from the second (top row) and the third (bottom row) MRI examinations. Several new subcortical white matter changes were found and are highlighted with yellow arrows. The discrepancy was not considered to be explained by the slight difference in image quality. All other sequences in all three MR examinations showed normal findings.

A previously healthy 40-year-old man developed mild respiratory symptoms without requiring hospitalisation. He had a positive nasopharyngeal swab for SARS-CoV-2. After 22 days, he developed acute psychotic symptoms, and within 1 day, he became mutistic and then unresponsive. Once admitted to the hospital, he showed a decorticated posture, rowing-eye-movements and bilateral corticospinal tract involvement with hyperreflexia and Babinski sign bilaterally as previously described (17). Standard morphological MRI was normal. Cerebrospinal Fluid (CSF) analysis showed pleiocytosis and no oligoclonal bands, and the test for antibodies to intracellular, synaptic or surface antigens was negative (Euroimmun kit, Luebeck, Germany). SARS-CoV-2 Polymerase Chain Reaction (PCR) in the CSF was negative. The discrepancy between the symptom severity and the negative MRI represented a diagnostic dilemma between early CNS infection and autoimmune encephalitis. Considering other cases of COVID-19-related encephalitis with normal morphological MRI (3, 9), the serious clinical picture, CSF pleiocytosis and recent history of SARS-CoV-2 infection, a treatment against suspected post-infectious autoimmune COVID-19-related encephalitis was started. The patient responded well to combined treatment with plasmapheresis and steroids. A second CSF analysis and a new MRI with DTI sequences were performed 11 days after neurological symptoms onset (33 days after first COVID-19 symptom). Pleiocytosis remained but was milder, and the patient developed oligoclonal bands selectively in the cerebrospinal fluid. The patient was discharged, 18 days after neurological symptom onset, remarkably improved with normal conventional neurological examination. Later laboratory analysis using indirect immunohistochemistry in brain tissue confirmed IgG autoantibodies against unknown targets that bind several brain areas with the highest level of immunoreactivity in neurons and neuropil in the caudate-putamen (17). At clinical follow-up, 68 days from neurological symptom onset, the neurological examination was normal, but both patient and his family described pronounced fatigability of the left leg, some difficulty recognising faces, increased anxiety and emotional lability. The patient underwent a follow-up MRI that showed new lesions in prefrontal lobes and bilateral sub-insular regions with right side predominance, but still no structural abnormalities of the corticospinal tract (Figure 1). The regional ethics committee approved the study protocol (Dnr 2012/081 and Dnr 2014/148). An informed consent from the patient included in this study was acquired.

### Control cohort

Ten healthy control individuals (HC) (mean age 26 ± 5; five females and five males) with no previous traumatic brain injury and neurological or neuropsychiatric conditions were included as a control group as previously reported (18, 19). The study was approved by the Regional Research Ethics Committee in Uppsala. A written informed consent was obtained from all included HC.

### Neuroimaging, DTI acquisition and processing

The patient was examined three times with the same protocol using a 3.0 Tesla scanner (Achieva dStream, Philips Medical System, Best, Netherlands). High-resolution 3D-T1-weighted (T1w), 3D-T2 fluid attenuated inversion recovery (3D-FLAIR), Diffusion-Weighed Imaging (DWI) and Susceptibility weighted imaging (SWI) were acquired for standard evaluation. The second and third scans included diffusion-weighted tensor images (DTI). Details about DTI acquisition, data processing and tracking technique are provided in Electronic supporting material 1 (ESM1).

**Figure 2:**
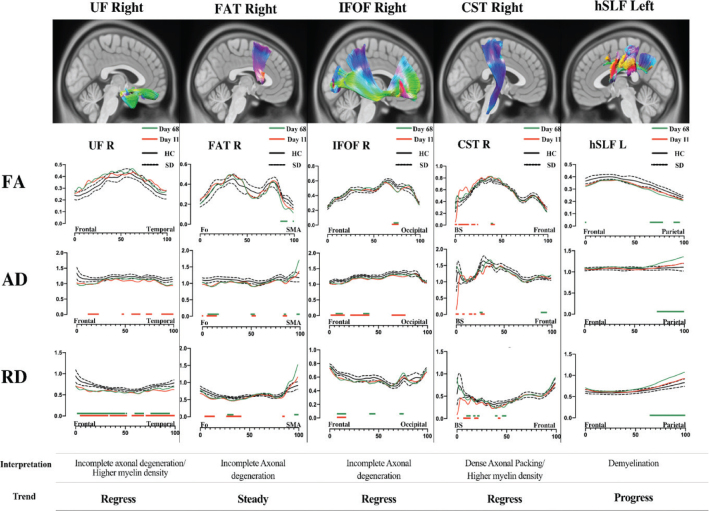
The analysis of the diffusion parameters for different white matter pathways is displayed. In the upper part, an illustrative picture of each white matter bundle with colours based on fibre directions. In the middle part of the images, the three graphs show the regional analysis of fractional anisotropy (FA), axial diffusivity (AD) and radial diffusivity (RD) at day 11 (red line) and at day 68 (green line) after the onset of neurological symptoms, in comparison with 10 HCs (mean values, black full line) and standard deviation (SD, black dotted line). On the axis line, separate red or green dots/lines indicate the significant difference (*P* < 0.05) at the Z test for each point analysed when compared with HC and corrected for multiple comparisons. False discovery rate was controlled using the Benjamini–Hochberg procedure with Q = 10% keeping the number of false positives below 10% of the number of significant indices. Only group of 11 sequential significant indices to indicate a significant difference compared to HC were displayed and interpreted as potential white matter abnormalities. Additional lobar/topographic regions are provided to indicate the localisation of the white matter changes. In the lower part of the image, an interpretation of the results is provided based on the combination of FA, AD and RD, and the interpreted trend between the investigations. SMA: supplementary motor area; Fo: frontal operculum; BS: brain stem.

White matter pathways from the patient were then compared to the HC average using a z-test for both examinations. Indices where the z-test reached a significance level of <0.05 are highlighted with red (day 11) and green (day 68) in Figure 2. The false discovery rate was controlled using the Benjamini–Hochberg procedure with Q = 10%. In this regard, the number of false positives is keep below 10% of the number of significant indices. Interpretations were made for each white matter track and diffusion parameter (FA, AD and RD) based on significant differences between day 11 compared to HC average and day 68 compared to HC average.

## Results

The first two MRI scans (day 2 and day 11 from neurological symptom onset) were negative (Figure 1, top row). When we compare the first DTI analysis (day 11) with the HC group, the right uncinate fasciculus (UF) and the cortico-spinal tract (CST) displayed focal signs of myelin changes compared with HC (high fractional anisotropy [FA], low axial diffusivity [AD] and low radial diffusivity [RD]). The right frontal aslant tract (FAT) and the right inferior fronto-occipital fasciculus (IFOF) displayed signs of axonal changes (low FA, low AD and low RD, highlighted in red in Figure 2). The other analysed white matter bundles did not show a significant difference compared with HC. In the third scan (day 68), several new, rounded subcortical white matter changes had appeared in frontal and sub-insular regions (predominantly in the right hemisphere) (Figure 1, bottom row). When the second DTI analysis (day 68) was compared with the first one and the HC group, we identified a partial regress of myelin changes in the right UF, the right CST and in the right IFOF. The right FAT showed steady focal axonal changes between investigations (at the compared AD abnormalities distribution), while the left horizontal component of the superior longitudinal fasciculus (hSLF) displayed new significant diffusion changes possibly reflecting myelin damage (low FA, high AD and high RD).

## Discussion

In this report, we present two main results. First, we confirm a limitation in the sensitivity of conventional MRI at the acute setting of SARS-CoV-2-related autoimmune encephalitis. As described by other reports, neuroimaging in COVID-19 is important to find haemorrhagic and thromboembolic lesions but is frequently negative despite neurological symptoms (4, 7, 8, 20). Neurological manifestations of COVID-19 disease have been extensively reported (1, 8, 16, 21), and therefore, any increase in the sensitivity of neuroimaging would be beneficial for our understanding of the disease.

Second, we detected early focal white matter changes using DTI while the conventional MRI was negative. DTI has been shown to be sensitive to even minor tissue changes not yet visible on conventional MR (10, 15). We were able to detect lower AD and RD compared with HC 11 days after encephalitis onset. In the earliest phase, AD/RD may be slightly reduced, similar to the observations in cerebral ischemia although less pronounced and without the subsequent development of necrosis in the affected area. Similar combinations of low AD/RD and higher FA have been reported in early stages of herpes simplex encephalitis and HIV infection (14, 15). Similar to the early ischemic phase, cell swelling due to cytotoxic oedema narrows the extracellular space, and thereby increases its tortuosity and anisotropy. The result is that a slightly increased FA could be considered as an indicator of cellular swelling due to possible viral intrusion and replication as supported by studies on other corona viruses (22–24). In later stages, an elevation of AD/RD and tissue swelling is expected, indicating inflammatory oedema with consequent lower FA as observed in other neurological diseases (15). In our patient, we observed an attenuation of diffusion changes in most of the pathways although a clear progression of changes in the left hSLF. These findings are partially in agreement with the only previous report on DTI findings in COVID-19 patients. Lu et al. demonstrated white matter microstructural changes with DTI in a cohort of patients, 3–4 months after COVID-19 infections (16). The FA was generally higher than controls, while AD and RD were generally lower compared with controls. We observed similar combination in our patient already at day 11 after the neurological symptom onset. However, possibly due to technical differences and study design, we detected a progress of white matter signals at day 68 in specific white matter pathways as a late consequence of inflammatory status (15, 25). Nevertheless, our results are based on a single case and should be carefully interpreted. As exemplified in this case, not all COVID-19 patients with neurological manifestations experience severe respiratory symptoms. Hence, a vascular-hypoxic damage to white matter cannot be the only etiological explanation, especially if structural lesions are not detected during the symptomatic stage (4, 5, 8, 9). Despite structural MRI sequences could not detect hypoxic/haemorrhagic damages and the patient had no history of neuropsychiatric or neurological diseases, without a brain biopsy, we are still unable to provide proof of exclusion of SARS-Cov-2 virus into the CNS (26) as a possible aetiology to the white matter abnormalities detected. On the other hand, the initial treatment for autoimmune encephalitis was effective confirming our hypothesis, and CSF-PCR for SARS-Cov-2 was negative in both lumbar punctures. In addition, the patient was neurologically and psychologically intact before COVID-19. The trend of different forms of regional white matter changes displayed by DTI analyses at early and subacute COVID-19 disease may suggest a stronger relationship between our findings and the course of COVID-19 disease. Therefore, we believe that the risk of neglecting other possible causes related to our findings is relatively low.

We formulate a pathophysiological hypothesis based on the clinical features, laboratory analyses and structural radiological investigations combining MRI and DTI. Our results suggest that the combination of conventional MRI and DTI could be a more sensitive diagnostic tool with high value for clinical neurologists in early therapeutic decisions of COVID-19-related neurological symptoms. In fact, in the modern era of neurology, clinicians are often reluctant to treat when standard morphological imaging does not reveal any structural lesions. This can lead to treatment delay, thus more severe symptoms and risk for sustained disability.

To improve our understanding of the pathophysiology of cerebral COVID-19 manifestations, only studies with larger cohorts of patients analysed with early and longitudinal MRI and DTI investigations would be able to better characterise lesion topography, temporal evolution of the lesions, development of symptoms and treatment response.

## Data Availability

The data that support the findings of this study are available on request from the corresponding author. The data are not publicly available due to privacy or ethical restrictions.

## Supplementary material

**ESM 1:** complementary description of imaging acquisition, data processing methods, DTI tracking methods used for the reconstruction of white matter bundles and related references.
